# Constipation

**Published:** 1899-01-14

**Authors:** 


### CONSTIPATION.

In a very interesting paper[1] which was lately read before the West Kent Medico-Chirurgical Society by Dr. Herschell, the subject of constipation is dealt with in a scientific spirit, and from its perusal many hints as to treatment may be gathered by those who care to go a step further in rational therapeutics than is usual with the ordinary vendor of drugs, who is satisfied if he can but knock down any symptom that stands in his way. Before turning to what he says about treatment it may be well to make note of two points on which he insists—first, that constipation is very frequently complicated, with retention of fæcal matter; in other words, that in addition to the slow passage of the contents of the intestines, which is characteristic of constipation, certain portions of them get, if one may say so, out of the direct current, and remain lodged for considerable periods, as practically foreign bodies; and, secondly, that in health the rectum should be empty, except when the act of defæcation is just about to be performed, the sygmoid flexure being the reservoir in which fæcal matter is stored until the time of the defæcation. We fancy that this point, namely, that fæces resting in the rectum is matter in a wrong place, is far too often overlooked.

Dr. Herschell arranges the abnormalities by which constipation may be produced as follows: 1. Defective peristalsis. (*a*) Due to absence of cellulose from the food. (*b*) Deficient irritability of the terminals of the nerves in the intestinal walls. (*c*) Muscular weakness of the intestinal walls. 2. Increased resistance. (*a*) From irregular peristalsis and spasm of portions of the bowel. This will include anal spasm. (*b*) Abnormal distension, with fæces or other materials. (*c*) Abnormal character of fæces, especially dryness. (*d*) Mechanical interference with the movements of the intestines; adhesions, pressure, stricture, or enteroptosis. The final act of defæcation may be defective from: 1. Abnormality or want of excitability of the spinal centre. (*a*) From local disease. (*b*) By inhibition from the brain or higher centres. 2. Weakness of the abdominal muscles. The diagnosis, then, of a case of constipation, or, in other words, the ascertainment of the cause of the condition, is a somewhat elaborate affair, and requires a very complete investigation into the history of the symptoms complained of, and into the physical conditions present.

In regard to treatment, Dr. Herschell gives the first place to a complete removal of all retained fæcal material from the intestines. This end he attains by the use of enemata, and prolonged irrigation if necessary, where the retention is low down, and by a succession of large douches where it is in the higher portions of the large intestine. In reference to these large douches, it is to be remembered that the bowel can only be induced to receive them by their being very steadily introduced, so that it is necessary to use a douche-can arranged at a proper level instead of the syringe. The pressure employed must be a low one, anything above two pounds to the square inch being dangerous to an adult, and much less to a child. For the purpose of ascertaining how things are going on during the operation, Dr. Herschell attaches a mercurial manometer to the tube. The temperature must be about 100 deg. F.; if either higher or lower the patient will suffer unnecessary pain. It must always be remembered that, when the retained fæcal matter has once been dislodged from its bed, the douches must be continued until it is satisfactorily removed, otherwise symptoms of auto-intoxication may be set up, a sort of ptomaine poisoning by the toxins set free from the old material.

To restore tone to the muscular and nervous tissues of the bowel, and to the abdominal wall, five means are practicable—hydrotherapy, electricity, massage and its substitutes, gymnastics, and drugs. In regard to hydrotherapy three measures may be mentioned: (*a*) Ice massage of the abdomen—that is the rubbing of the abdomen with a block of ice following a hot bath; (*b*) the cold abdominal douche, the patient lying in a bath in warm water (but with the abdomen bare), an attendant pours, from a height of two or three feet, a jug of water upon the abdomen; (*c*) the cold spinal douche; the patient kneeling in a tub containing a little warm water and leaning slightly forward. Jugs of cold water are then poured down the back. If hydrotherapy is to be effectual there must be a contrast or an alternation; the whole body must not be made either hot or cold. In using electricity the continuous galvanic current is employed to restore the tone to the nervous supply of the intestines; a special faradic coil, the primary coil of which is wound with very thick wire, such as was introduced by Dr. de Watteville for use in the faradic bath, is used to restore tone to the musculature of the intestines; while the ordinary faradic secondary current is used to strengthen the abdominal muscles. Massage appears to be not altogether without its dangers, and in place of it Dr. Herschell uses a particular form of vibrator, which, we believe, he exhibited at the Edinburgh meeting of the British Medical Association.

Gymnastics for the abdominal muscles are very

[1] Clinical Journal, Nov., 1898.

important in the treatment of constipation. It would be easy to write a considerable paper on this subject alone, but a little careful thought as to the movements which bring the abdominal muscles into play will indicate the sort of exercises to be used. The best time is immediately on rising in the morning, and again on retiring at night. It is worth bearing in mind that cycling is of great service in relieving some forms of constipation. The regulation of diet, again is a large subject; but all we can do here is to point out that food may be laxative, because (1) it may contain a considerable amount of cellulose, a most important ingredient; (2) it may contain purgative salts or sugar, as in prunes, apples, &c.; (3) it may contain hepatic stimulants, as in the dandelion; (4) it also may be laxative in consequence of being rich in fat. If, then, the stomach is in good order, so as to be able to digest any sort of food, it is not difficult to arrange a fairly laxative diet. Unfortunately, the stomach often stands in the way, for many of the kinds of food which are most useful are just what the stomach cannot cope with. Thus, indigestion or feeble digestion becomes a fertile cause of constipation. Bran is often one of the most useful remedies for constipation due to lack of cellulose. It may be taken stewed with apples, or in the form of biscuits, or even in cachets.

Finally, as to the use of drugs. Many kinds are of great service. Those which excite peristalsis, or relieve spasm, or increase the secretions of the gastro-intestinal tract are of much service, also tonics of various sorts, but as to mere laxatives they are only to be used in special circumstances. Practically, the only cases in which it is good practice to have recourse to a daily laxative drug are those in which the stomach is so dilated or atonic that a diet appropriate for the constipation cannot be arranged. It may, however, be necessary, to give enemata either of oil or of cold water to maintain the action of the bowels during treatment, until the constipation shall have been otherwise overcome.

